# Selective condensation of DNA by aminoglycoside antibiotics

**DOI:** 10.1007/s00249-015-1095-9

**Published:** 2015-12-08

**Authors:** M. Kopaczynska, A. Schulz, K. Fraczkowska, S. Kraszewski, H. Podbielska, J. H. Fuhrhop

**Affiliations:** Department of Biomedical Engineering, Faculty of Fundamental Problems of Technology, Wroclaw University of Technology, Wybrzeże Wyspiańskiego 27, 50-370 Wrocław, Poland; Institut für Chemie and Biochemie, Organische Chemie, Freie Universität Berlin, Takustr. 3, 14195 Berlin, Germany

**Keywords:** Deoxyribonucleic acid condensation, Toroids, Aminoglycoside antibiotics, Atomic force microscopy, Electron microscopy, Molecular dynamics

## Abstract

The condensing effect of aminoglycoside antibiotics on the structure of double-stranded DNA was examined. The selective condensation of DNA by small molecules is an interesting approach in biotechnology. Here, we present the interaction between calf thymus DNA and three types of antibiotic molecules: tobramycin, kanamycin, and neomycin. Several techniques were applied to study this effect. Atomic force microscopy, transmission electron microscopy images, and nuclear magnetic resonance spectra showed that the interaction of tobramycin with double-stranded DNA caused the rod, toroid, and sphere formation and very strong condensation of DNA strands, which was not observed in the case of other aminoglycosides used in the experiment. Studies on the mechanisms by which small molecules interact with DNA are important in understanding their functioning in cells, in designing new and efficient drugs, or in minimizing their adverse side effects. Specific interactions between tobramycin and DNA double helix was modeled using molecular dynamics simulations. Simulation study shows the aminoglycoside specificity to bend DNA double helix, shedding light on the origins of toroid formation. This phenomenon may lighten the ototoxicity or nephrotoxicity issues, but also other adverse reactions of aminoglycoside antibiotics in the human body.

**Electronic supplementary material:**

The online version of this article (doi:10.1007/s00249-015-1095-9) contains supplementary material, which is available to authorized users.

## Introduction

Genetic information is accumulated in a double helix of DNA. Since genomic DNA is a very long molecule, it must be condensed to fit into a small space of cell nucleus or a virus particle. Nevertheless, it must be readily accessible for various activities such as replication and gene expression. Therefore, condensation of DNA chains is a fundamental life process, and it has been the subject of many studies over the last decades (Wilson and Bloomfield 1979; Ma and Bloomfield 1994; Bloomfield 1991, 1996, 1997; Saccardo et al. 2009; Nayvelt et al. 2010; Teif and Bohinc 2011; Lee et al. 2014). In human cells, DNA with a length of order of 2 m is packed in the nucleus, which has a diameter of about 5 µm. Nucleic acids in solution are highly charged biopolymers. This fact considerably complicates tight packing of DNA due to the repulsion between strands. In somatic cells, DNA of the human genome is wrapped around positively charged histone octamers and packed into chromosomes (O’Donnell et al. 2013; Hirano 2014).

DNA condensation is also observed in vitro by inducing attractive interactions between chains, or applying an external force, which brings the double helices together (Bloomfield 1996, 1997; Teif and Bohinc 2011). The formation of condensed structures may be realized by reducing the repulsion between the DNA chains by multiple agents such as multivalent inorganic cations (Bloomfield 1997), lipids and liposomes (Rao 2010), metal ions (Li et al. 2011), protamines (Balhorn 2007), polyamines (Nayvelt et al. 2010), nanomaterials (An and Jin 2012), and peptides and proteins (Saccardo et al. 2009). The second possibility may be caused by the osmotic pressure derived from neutral crowding polymers in the presence of monovalent salts as a result of the entropic random collisions of DNA with the surrounding polymers. The presence of salt is required to neutralize DNA charge and decrease the electrostatic repulsion of the negatively charged DNA phosphates (Teif and Bohinc 2011). The external factors induce a condensation phase transition of DNA. As a result of this process, extended DNA duplexes aggregate into compact, ordered structures, consisting of several DNA strands. The condensed structures of DNA observed in vitro are greatly similar to these noticed in vivo in viruses and in sperm cells. Experimentally, toroidal and rod-like shaped forms are the most commonly observed structures, but also oval, spherical, disk-like, and flower-like aggregates are noticed. The morphologies of condensed DNA strands and their occurrence frequency are conditioned by many factors, including size and charge of the condensing agents, molecular structure, the amine-to-phosphate ratio, salt concentration, and valence (Zhou et al. 2013).

Studies of DNA condensation have opened new perspectives in medicine and biotechnology. The main potential application of nucleic acid complexes is gene delivery into a patient’s cells by viral or non-viral vectors in gene therapy (Kotterman and Schaffer 2014; Lee et al. 2014). Furthermore, condensation of nucleic acids by ligands is used in the treatment of various diseases, especially cancers. Several drugs act through cross-linking, intercalating DNA molecules, electrostatic interactions or binding to minor/major groove of helices, and establishing condensed untranscribed structures (Rauf et al. 2005). On the other hand, condensation of DNA may be an undesirable process. Drugs and other molecules delivered to the human body can cause side effects by interaction with genomic DNA. For example, ergot derivatives have a direct cytotoxic effect on astrocytes and renal proximal tubule epithelial cells with noticeable DNA condensation and fragmentation (Mulac and Humpf 2011). The actions of ligands on DNA lead to the inhibition of biological activity of nucleic acids, disruption of protein biosynthesis, and eventually to cell death. Moreover, binding of drugs to nucleic acids has important consequences for certain diseases such as cystic fibrosis (CF). Patients with CF are more susceptible to respiratory bacterial infections as a result of increased accumulation of viscous mucus in the airway. These infections are treated with inhaled antibiotics such as aminoglycosides. Lysed inflammatory cells release DNA, which is found at high concentrations (<20 mg/ml) in the mucus layer. The DNA molecules bind to and severely reduce the activity of cationic aminoglycosides (Drew et al. 2009).

Aminoglycoside antibiotics (e.g., kanamycin, tobramycin, neomycin) are low molecular weight molecules (300–600 Da). They contain a cyclitol ring linked to five- or six-membered sugars by glycosidic bonds (Nikolaus and Strehlitz 2014). The whole structure contains several free hydroxy and amino moieties. In biological media, the amine groups are mainly protonated, therefore aminoglycoside antibiotics may be considered as polycationic species in order to understand their biological action. Due to the positive charge of the molecule, they show a binding affinity to nucleic acids (Kotra et al. 2000).

Aminoglycosides are important antibiotics for the treatment of various infections caused by Gram-negative and some of Gram-positive bacteria (Hermann 2007). The main bactericidal mechanism of action of aminoglycosides is the inhibition of protein synthesis. They bind to the prokaryotic 30S ribosomal subunit, hence the ribosome is unavailable for translation. Other bactericidal activity of aminoglycosides includes disintegration of the cell membrane, altered cellular ionic concentrations, and impaired synthesis of RNA and DNA. A crucial step before they bind to the ribosome is an active drug transport into the bacterial cell, which depends on oxygen and energy (McCollister et al. 2011). Cellular uptake of aminoglycosides in bacteria requires proton motive force (PMF), generated by electron flow through the respiratory chain (Ezraty et al. 2013). This transport is inhibited by divalent cations, under anaerobic conditions, and at low pH (Jana and Deb 2006).

Despite the high antibacterial efficacy, all aminoglycosides can cause adverse side effects, especially, in relation to the kidneys and the inner ear. While the nephrotoxicity inflicted by these antibiotics is usually reversible, ototoxicity is permanent. Several studies have indicated that gene mutations are involved in the aminoglycoside toxicity (Dehne et al. 2002). Their serum clearance is rapid (<24 h), but they are preferentially accumulated and maintained in proximal tubule cells of the kidney and hair cells in the cochlea of the inner ear. In hair cells, aminoglycosides are poorly degraded and have long half-lives, approximately 5–6 months (Steyger et al. 2003). The precise mechanism of their toxicity is still not fully understood. The cellular uptake of aminoglycosides has been extensively studied. Previous studies using electron microscopy demonstrated the evident distribution of the aminoglycoside antibiotic—gentamicin into the cytoplasmic compartments of mammalian tubule cells (Gilbert et al. 1989). The intracellular uptake of aminoglycosides was also observed in sensory hair cells (Steyger et al. 2003). When aminoglycosides penetrate the cell, they may interact with intracellular components such as nucleic acids and cause the toxicity. It is suggested that both endocytosis and transport through various membrane transporters contribute to the uptake of these drugs into mammalian cells (Marcotti et al. 2005; Denamur et al. 2008).

In the present study, the interactions of three aminoglycoside antibiotics such as tobramycin, kanamycin, and neomycin with calf thymus DNA were examined using high-resolution nanoscopic techniques such as NMR spectroscopy, atomic force microscopy (AFM), and transmission electron microscopy (TEM). To the best of our knowledge, this is the first attempt to study at the nanoscale the direct impact of aminoglycoside antibiotics on structural changes of DNA. Understanding of these interactions may explain many problems associated with severe side effects of treatment with using these antibiotics such as ototoxicity or nephrotoxicity. Here, we report the rod, toroid, and sphere formation and very strong condensation of DNA molecules by tobramycin, which was not observed in the case of other aminoglycosides used in the study. The effect of interaction between aminoglycoside antibiotics and DNA strongly depends on the hydration sphere of the deoxyribose diphosphate part of DNA and that of a deoxyglucose unit of an aminoglycoside. Obtained results are discussed in context of molecular mechanism of interactions derived from theoretical molecular dynamics (MD) studies on the B-DNA–tobramycin complex.

## Materials and methods

### Materials

Neutral, lyophilized form of kanamycin, tobramycin, neomycin and calf thymus DNA (ct-DNA, ~4000 and 8000 bp), deuterated solvents for NMR spectroscopy studies and staining materials for electron microscopy studies were purchased from Sigma.

### ^1^H NMR spectroscopy

**Fig. 1:**
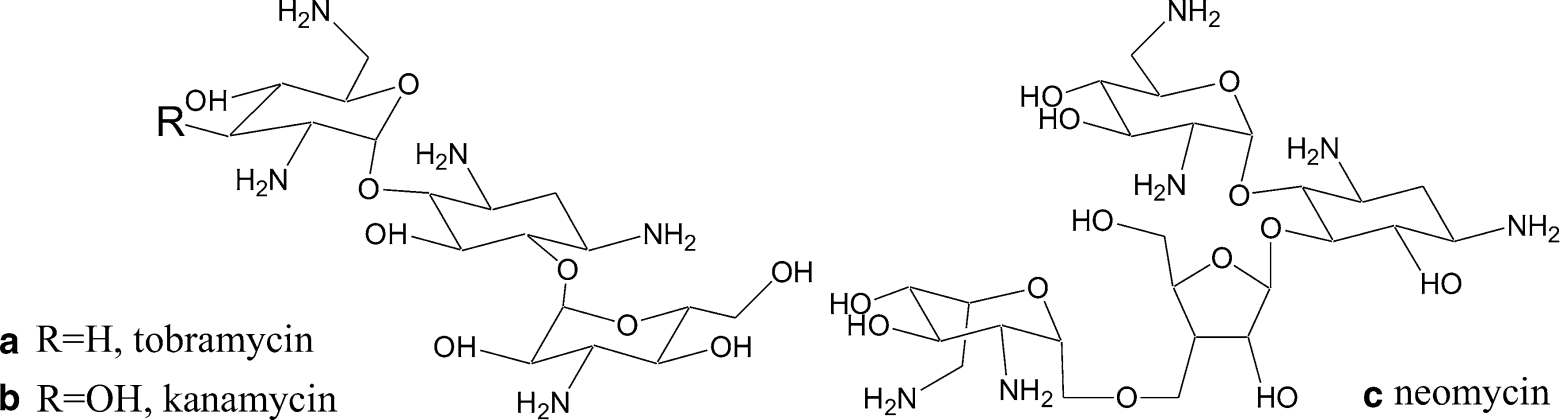
Scheme of the aminoglycoside antibiotics used in the study. **a** Tobramycin, **b** kanamycin, **c** neomycin

^1^H NMR spectra were obtained with a Bruker AMX 500 spectrometer operating in the quadrature mode at 500 MHz. The residual peaks of deuterated solvent D_2_O were used as internal standards. NMR spectroscopy was done with milligram quantities of calf thymus DNA and the natural aminoglycosides: tobramycin (Fig. 1a), kanamycin (Fig. 1b), and neomycin (Fig. 1c). ^1^H NMR spectra of each drug in D_2_O solution have been recorded. Afterwards, 1 mg of tobramycin, kanamycin, or neomycin in 1 ml of D_2_O were mixed with 1 mg of DNA corresponding to a base pair-to-aminoglycoside ratio of about 1.0. The samples were subsequently measured in D_2_O solution.

### AFM measurement

The DNA probes without and with added aminoglycosides were imaged by atomic force microscopy (AFM) using a MultiMode IIIa scanning probe microscope with the extender module (Digital Instruments, Inc., Santa Barbara, CA, USA) that was operated in the dynamic modus and a MultiMode IIId scanning probe microscope (Veeco Instruments GmbH, Mannheim, Germany). Height measurements of the objects were applied with an instrumental vertical resolution of ±0.1 nm. Olympus etched silicon cantilevers were used with a typical resonance frequency in the range of 183–192 kHz and a spring constant of 43 N/m. The set-point amplitude of the cantilever was maintained by the feedback circuitry to 80 % of the free oscillation amplitude of the cantilever. The scan angle was maintained at 0°, and the images were captured in the trace direction with a scan rate between 0.500 and 1.500 Hz. All samples were measured at room temperature in air. The sample was first adjusted with an optical light microscope (Nanoscale, Optical Viewing System). Data analysis was performed after flatten and plane-fit auto, height measurements based on the cross-sectional profiles and/or analysis. Nitrogen gas flow was applied on the unmodified mica surface for alignment and separation of DNA molecules.

For AFM experiments, calf thymus DNA was used. Commercial mica platelets (Plano) were used as subphase. Calf thymus DNA was dissolved in buffer containing 20 mM of HEPES, 150 mM KCl, and 150 mM MgCl_2_, pH 7.4. The Mg^2+^ ions were needed for the deposition of DNA on the mica surface (Podestà et al. 2005). Next, a 20-µl droplet of antibiotic (kanamycin, tobramycin, or neomycin) in Milli-Q water (pH 7) was placed at the same place on the mica surface. The ratios aminoglycoside/DNA were 0.2:1, equimolar (1:1) and 10:1. Any excess fluid was blotted off after 10 s.

### TEM measurements

DNA with or without aminoglycosides was imaged with a Philips CM12 transmission electron microscope operated at 100 kV. High-resolution transmission electron microscopy (TEM) was performed on the calf thymus DNA. A 10-µl droplet of DNA solution was placed onto a grid covered first with a collodion film, and then with carbon vapor to render the carbon powder layer immobilized under an argon stream. Then, after 2 min of residence time, the argon gas was applied to the sample with a pressure of about 0.5 bars. The distance between the grid and the gas hose outlet was kept constant at about 1 cm with an angle of approximately 45°. The incubation time was 2.5 min. Measurements were then performed without or with staining solutions containing 1 % of uranyl acetate or phosphotungstate, which was blotted off after 60 s. The samples with aminoglycosides were prepared analogously as in the case of AFM study.

### Molecular dynamics studies

Different systems were tested by means of MD during an overall simulation time reaching 0.45 μs. System 1 (S1) concerns the 12 base pairs B-DNA fragment (CGCGAATTCGCG) and serves here as a reference. In S1, B-DNA was hydrated with a solution containing water molecules and 150 mmol/l dissociated NaCl in the simulation box of about 195 nm^3^. S1 was run for 35 ns. Since the equilibrium state is quickly obtained for S1 and for comparison purposes, we restarted S1 from the beginning and we added one tobramycin molecule, which was initially located 2 nm from the B-DNA surface, thus creating the S2 system. From this configuration, we proceeded to a 169-ns production run. S2 is used to study the binding sites of tobramycin onto B-DNA. The binding of multiple tobramycin molecules onto B-DNA has also been studied through the S3 system. In this case, we have added to the initial state of S1 three tobramycin molecules and performed a 136-ns production run. The tobramycin molecules were initially placed randomly in the resulting MD box and far from the B-DNA (at least 2 nm from B-DNA surface, and 3 nm between them). Finally, supposing the cooperative effect of aminoglycoside antibiotics, we created the system S4 with four tobramycin molecules alone in the same hydration box size as for S1. S4 was run for 106 ns in order to elucidate tobramycin’s ability to aggregate.

All molecular dynamics (MD) simulations were carried out in the NPT (constant number of particles, pressure, and temperature) ensemble using the NAMD 2.9 suite of programs (Phillips et al. 2005). Langevin dynamics and Langevin piston methods were applied to keep the temperature (300 K) and pressure (1 atm) of the system constant. Long-range electrostatic forces were taken into account using the particle-mesh Ewald approach (Darden et al. 1993), and the integration time step was equal to 2 fs. B-DNA was described using the all-atom CHARMM27 force field known for proper reproduction of nucleic acid behavior in a water environment (MacKerell et al. 1998). The water molecules were treated using TIP3P model (Jorgensen et al. 1983). All the necessary potential parameters of the tobramycin molecule were derived by our quantum mechanics (QM) calculations, and we followed the general procedure described by Norrby and Brandt (2001). All quantum level calculations were performed using the Gaussian 09 software package (Frisch et al. 2013). The ground-state equilibrium geometry of tobramycin was optimized by density functional theory (DFT) model with the b3lyp/6–31 + g basis set (Hohenberg and Kohn 1964; Kohn and Sham 1965). The water solvent effect, which is crucial for charge screening, and thus for appropriate molecule hydrophilicity reproduction, was taken into consideration using the integral equation formalism of the polarizable continuum model (IEFPCM) with a dielectric constant *ε* = 78.39 (Tomasi et al. 1999). ESP partial charges were derived, since they are commonly assumed as compatible with CHARMM force field.

## Results

### ^1^H NMR spectroscopy

**Fig. 2:**
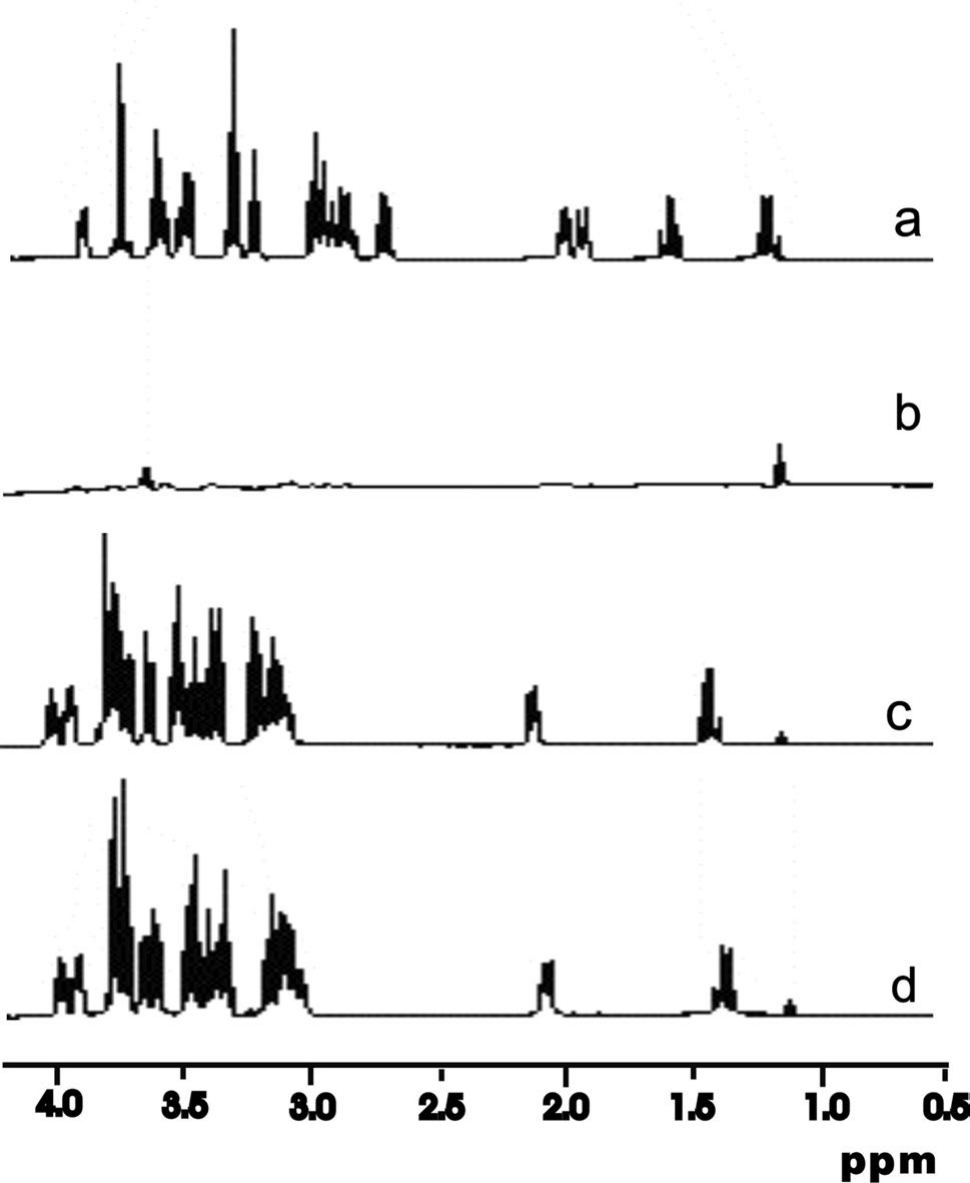
^1^H-NMR spectra of tobramycin (**a**, **b**) and kanamycin (**c**, **d**) (1 mg/ml) in the absence (**a**, **c**) and in the presence (**b**, **d**) of calf thymus DNA (1 mg/ml)

The first step of our research was to perform NMR spectroscopy with calf thymus DNA and aminoglycosides. ^1^H NMR spectra of each drug in D_2_O solution before (Fig. 2a, c) and after (Fig. 2b, d) addition of DNA have been recorded. The study was performed in order to observe any changes in NMR signal after reaction of DNA with aminoglycosides. We noticed an interesting phenomenon. In the case of tobramycin with DNA, the NMR signal has almost completely vanished (Fig. 2b). Furthermore, a white precipitate appeared in the solution, which may indicate aggregate formation. On the other hand, kanamycin and neomycin NMR signal intensities did not change after the addition of DNA (Fig. 2d). The DNA solutions containing kanamycin or neomycin were transparent and remained clear even with a tenfold excess of the latter.

### Morphology of DNA molecules with tobramycin

**Fig. 3:**
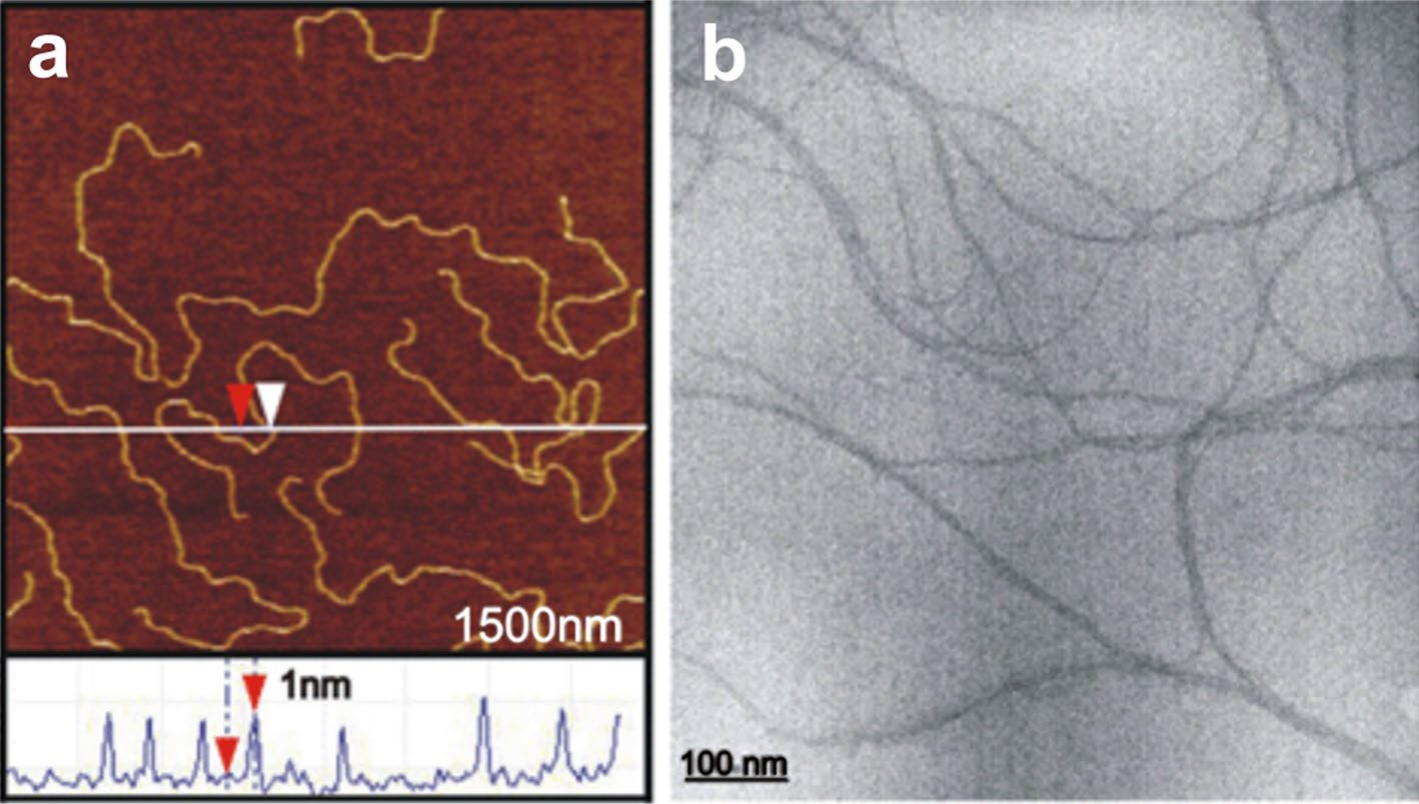
**a** AFM and **b** TEM images of native ct-DNA

In order to observe the DNA morphology after administration of aminoglycosides, microscopic techniques were used. AFM and TEM imaging were performed with stretched calf thymus DNA on mica and calf thymus DNA on carbon-coated grids, respectively. Figure 3 shows the representative AFM (Fig. 3a) and TEM (Fig. 3b) images of DNA. Uniform linear strands with a height of about 1.0 nm, corresponding to 50 % of the original double helix, were obtained (Fig. 3a). Although the sample has hydrophilic properties, DNA did not show a tendency to condense on either the hydrophilic (Fig. 3a) or the hydrophobic (Fig. 3b) surface. DNA chains were loosely scattered all over the surface and took a variety of curvatures—straight and bent with different degrees of bending.

**Fig. 4:**
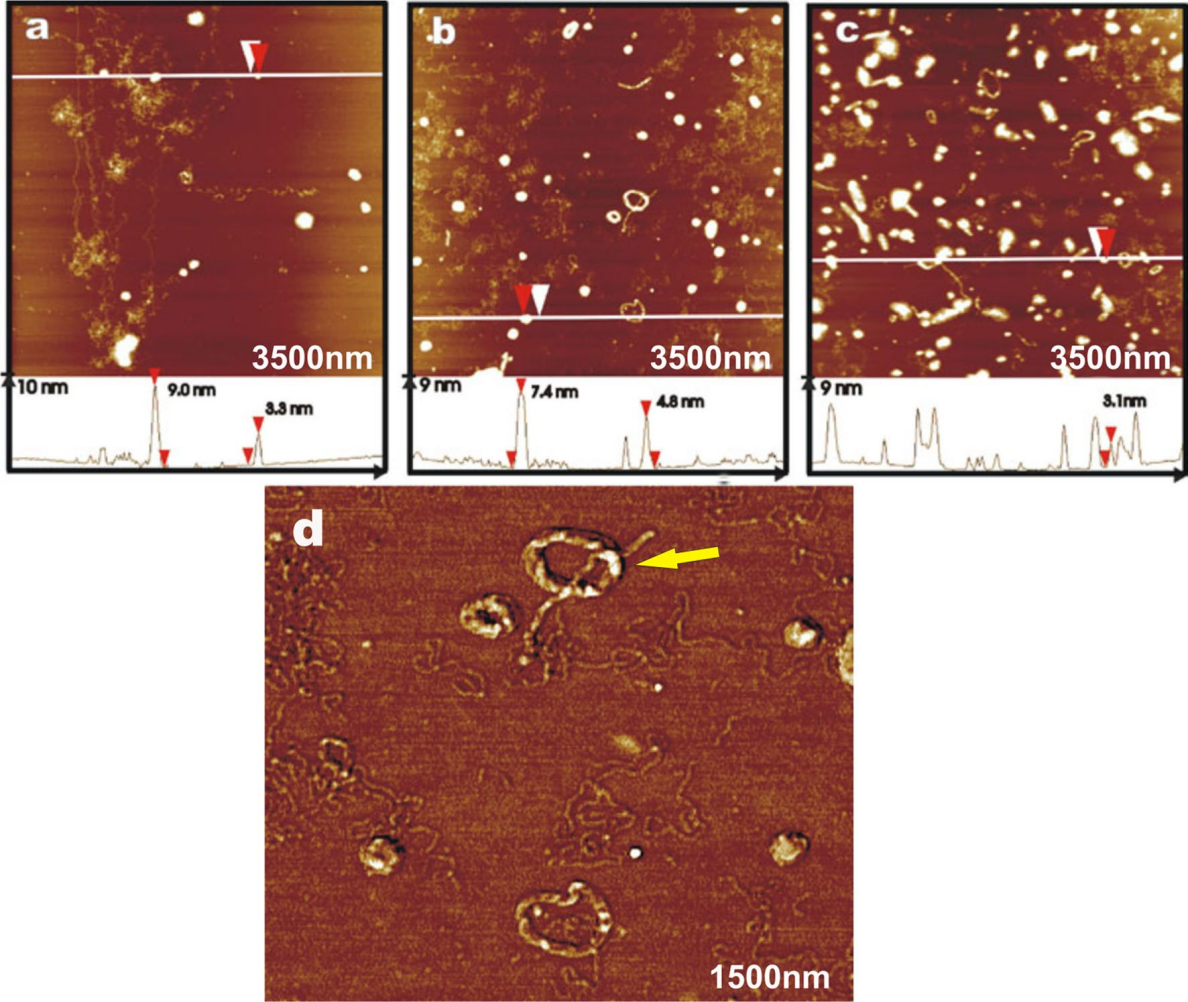
AFM images of DNA on mica after the addition of tobramycin with different concentrations. The ratios of tobramycin:DNA were **a** 0.2:1, **b** equimolar (1:1), and **c** 10:1. **d** AFM phase image of condensed DNA structures on the mica surface. *Yellow arrow* indicates toroid as a result of annularization of rod-like structure

Figure 4 shows the data obtained in the presence of tobramycin in 0.2:1, 1:1, and 10:1 tobramycin:DNA base pair molar ratios. At low tobramycin concentration, DNA began to condense to compact structures, however some of the DNA chains were elongated and still distinguishable (Fig. 4a). A further increase of drug concentration resulted in condensation of DNA and an extensive formation of more or less compact structures such as toroids, spheres, and rods was exclusively observed (Fig. 4b). In addition, intermediate structures were also present. The condensed forms were scattered on the surface of mica. Moreover, DNA chains in the background were entangled and coiled and tended to aggregate. The highest tobramycin concentration caused massive aggregation of nucleic acids into compact spheres, rods, and toroids with almost unnoticeable single DNA molecules (Fig. 4c). Results show that low tobramycin concentration produces about 15 % toroids, equimolar amounts about 50 %, and an excess of drug results in a quantitative yield of toroids. From the concentration of tobramycin and the observed toroid/linear strand ratio of the DNA, an equilibrium constant of *K* = *c*(base pairs) × *c*(tobra)/*c*(base pair-tobra) = 5 × 10^3^ was calculated. We also performed AFM phase imaging of sample in an equimolar drug:DNA ratio (Fig. 4d). The image shows a zoom of condensed structures—toroids, rods, and spheres.

**Fig. 5:**
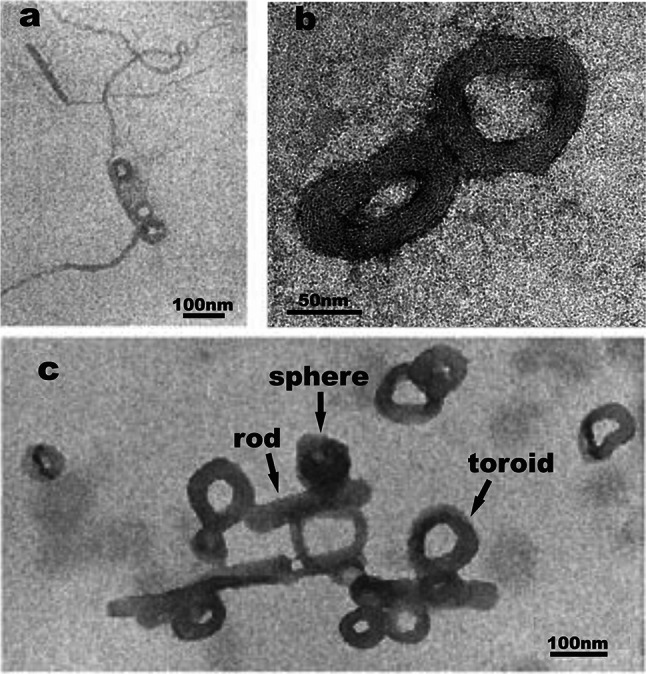
TEM images of DNA on carbon-coated grid after the addition of tobramycin in ratios of tobramycin:DNA **a** 0.2:1, **b** high-resolution image of ratio 1:1, and **c** 10:1. Labeled forms of DNA condensation: toroid, sphere, and rod-like structure

In order to compare the above-mentioned observations with results obtained by another technique, TEM examination of DNA–tobramycin interaction was performed. TEM images of DNA on carbon-coated grids with 0.2:1, 1:1, and 10:1 ratios of tobramycin per base pair, without any stain (Fig. 5), showed similar structures as in the case of AFM. When the concentration of drug increased, DNA became more and more condensed until complete DNA collapse occurred. At low tobramycin concentration, we observed a formation of toroids and rods (Fig. 5a). When the concentration of drug is higher, toroids turned into more compact spheres (Fig. 5c). High-resolution TEM image (Fig. 5b) illustrates the toroidal structure of condensed DNA, with a noticeable hole in the center surrounded by circumferentially wound strands.

**Fig. 6:**
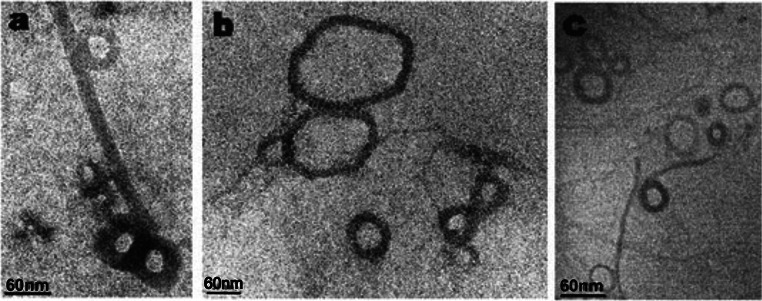
TEM images of toroids with 1 % phosphotungstate (**a**), 1 % uranylacetate (**b**), and without staining solution (**c**)

In order to enhance the contrast, we prepared samples stained with heavy metal salts, 1 % phosphotungstate (Fig. 6a) and 1 % uranylacetate (Fig. 6b). TEM images showed the same toroidal and rod-like structures. We compared these results with imaging without any staining materials (Fig. 6c). There is no obvious impact of the heavy metal staining on the morphology of DNA.

### Morphology of DNA molecules with kanamycin and neomycin

**Fig. 7:**
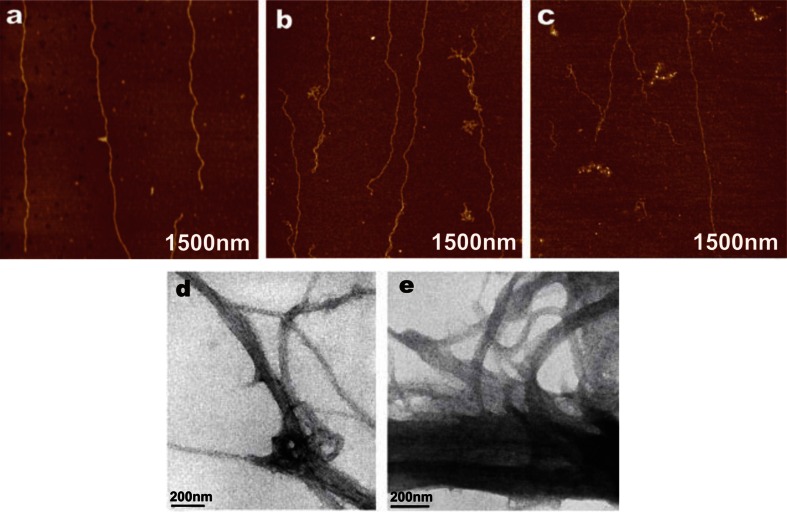
AFM images of native calf thymus DNA with the addition of kanamycin (**a**), with addition of neomycin (**b**), and on mica at equimolar-to-base pairs ratio (**c**). TEM images of calf thymus DNA with the addition of kanamycin (**d**) and with addition neomycin (**e**) in the same conditions

Further on, we performed a study on interactions of kanamycin and neomycin with DNA molecules by means of AFM and TEM imaging. These aminoglycosides did not show such effects as in the case of tobramycin. In drug–DNA 1:1 concentration ratio, no self-assemble process or toroid formation were detected. Nevertheless, we observed other intriguing phenomenon. In both cases, structural distortions and double-strand breaks were observed (Fig. 7). DNA forms are not distinguishable after addition of both kanamycin (Fig. 7b) and neomycin (Fig. 7c). As compared to the image of DNA with no drug (Fig. 7a), under the influence of examined aminoglycosides, DNA chains were cleaved and some of them entangled and aggregated without ordered structures. Figure 7d, e illustrate the results obtained by TEM of kanamycin and neomycin interactions with DNA, respectively. There is no toroid formation in any case, but some degradation of DNA fibers is observed.

### Molecular mechanism of interactions between double helix DNA and tobramycin

**Fig. 8:**
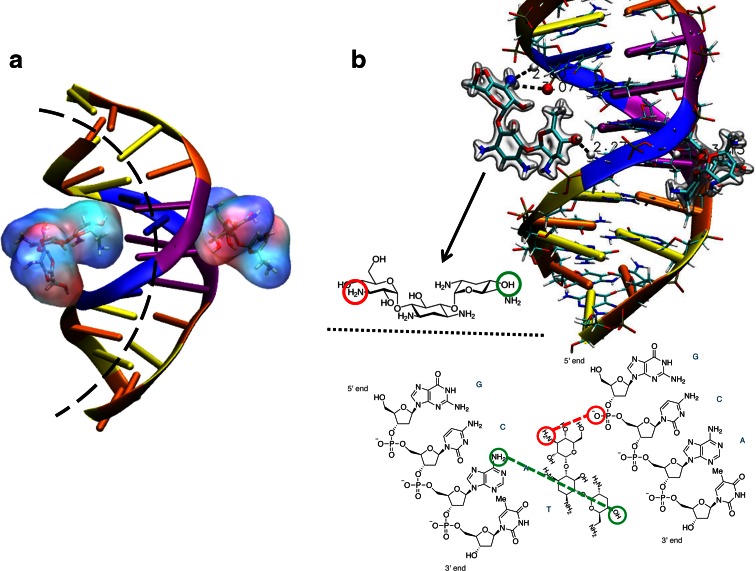
Molecular mechanism of tobramycin interaction with DNA. **a** Two aminoglycosides acting as a clamp induce DNA curvature. **b** Zoom on hydrogen bonding between specific hydrogen of tobramycin molecule and oxygen of deoxyribose 5-phosphate

Molecular dynamics simulations were performed in order to determine the specific interactions between tobramycin and DNA chain, which are at the origins of unusual forms of DNA condensation. From the studied models, we primarily found that tobramycin molecules do not exhibit any tendency to form aggregates between them under applied conditions, which is in an agreement with our previous studies assuming the lack of aggregation pH 7 (Kopaczynska et al. 2004). When a single tobramycin molecule appears in the vicinity of the DNA helix, our model shows strong but unspecific interaction between them with the energy binding of −87.6 ± 11.3 kcal/mol manifested as the aminoglycoside sliding along DNA groove. In detail, electrostatic component of −90.35 ± 9.73 kcal/mol strongly dominates over repulsive van de Waals (VdW) component (+2.75 ± 1.53 kcal/mol), showing the hydrophilic character of the studied antibiotic. When multiple tobramycin molecules are present in the direct neighborhood of DNA they start acting in pairs as a clamp (Fig. 8a). A similar mechanism was already observed for Hoechst 33258 dye (Utsuno et al. 2002). Antibiotic concentrations tested in MD simulations were 1:12 (tobramycin:base pairs) and 1:4 molar ratio, respectively, and we are aware that they may not be directly compared to the experiment due to the short length of B-DNA double helix used, unable to reproduce the experimentally observed condensation. However, we tempted to predict DNA curvature change resulting from tobramycin binding. Defining the bend angle as the angle of normal vectors of the first and last base pairs plains, we found that free DNA double helix with given bases sequence is dynamically bend with the angle 1.9° ± 1.0° per base pair, while binding of the first tobramycin molecule induces the higher bend angle of 2.5° ± 0.8° per base pair with restriction of DNA dynamics seen in standard deviation decrease. Surprisingly, further binding of tobramycin does not change this value more than 3 %. Even if the change in bend angle seems modest, we attract the reader’s attention to the fact that to form a full circle with tobramycin/B-DNA complex only 13.7 ± 3.3 helical turns are necessary (assuming 10.5 base pairs per helical turn), while one will need 18.0 ± 20.0 helical turns to form an energetically stable DNA full circle. In the second case, an uncurved DNA structure can also occur, according to experiment, due to standard deviation bigger than the value itself, and giving the nonzero probability for global bend angle of 0° (uncurved but gently writhed overall DNA helix). Finally, since the tobramycin molecule differs from kanamycin only by one molecular group, –H instead of –OH as shown in Fig. 1, we decided to monitor this particular hydrogen over molecular trajectory dynamics. Most of the time, this hydrogen tries to form a hydrogen bond with oxygen from deoxyribose 5-phosphate as shown in Fig. 8b. On the contrary, –OH groups, present in a greater number in tobramycin molecules than –H one, do not exhibit any long-lasting hydrogen bonding with DNA backbone. What we can precisely observe from MD simulations (as shown in Supplementary Information video) is that tobramycin molecules are attacking DNA through minor and then through major grooves. In minor groove case, two Tobra OH groups from both side ring and central ring decrease DNA dynamics (thermal fluctuations) through interactions with CH groups of DNA, probably in random pattern. In major groove case, OH group from other tobramycin molecule is interacting with one chosen NH group of purine base forming hydrogen bond. NH group from the other side of this second tobramycin molecule is interacting with phosphate oxygen from the third nucleic acid in the second chain, thus inducing strong DNA bend. The most surprising observation is that the second tobramycin molecule (the one attacking major groove) is choosing its position in front of first tobramycin molecule bound in minor groove. We could speculate that specific interactions of that molecule in minor groove are at the origins of electrostatic well formation in major groove on the other side of DNA helix. However, the reader should be aware that the observed situation is highly dynamic and that described configuration is not lasting for a long time over 100 ns. This could explain the very low concentrations of tobramycin molecules already precipitating DNA in toroid form.

We would emphasize the fact that there is no straight relation between microscopic DNA aggregates shown in the experiment and the molecular mechanism unrevealed on single and short DNA helix using numerical simulations. However, taking into account atom-level driving forces inducing small local changes in DNA curvature, we strongly believe that the proposed molecular mechanism may underline to the origins of unusual DNA aggregation, explaining the observed toroid forms. Thus, Fig. 8 could be treated as an up-to-atoms highly zoomed tiny part of microscopic DNA aggregate.

## Discussion

In this work, we present for the first time a comparative in vitro study of the effects of three natural aminoglycoside antibiotics—tobramycin, kanamycin, and neomycin on the tertiary structure of DNA. Studies on the mechanism by which small molecules bind to DNA have been identified as one of the crucial issues. In addition, it is of great interest to understand the structural properties of DNA, the mutation of genes, the mechanism of functioning of some drugs in the living cells, and their toxic effects at the molecular level. In order to examine the drug–DNA interactions, we used three different techniques, such as ^1^H NMR spectroscopy, atomic force microscopy (AFM), and transmission electron microscopy (TEM), supported by molecular dynamics simulations.

We have observed significant differences in the molecular actions of examined aminoglycosides on DNA conformation. The ^1^H NMR spectra obtained in this study suggest that tobramycin binds to and induces condensation of DNA. This phenomenon is evidenced by the fact that after the addition of tobramycin to DNA, a white precipitate was formed, which suppressed the signal completely. Remarkably, kanamycin and neomycin did not show the above-described effects and the signal remained unchanged even with a tenfold excess of the drug comparing to base pairs.

For a deeper understanding of aminoglycoside—DNA interactions, we performed AFM and TEM imaging. The application of AFM and TEM techniques in studying nucleic acid—ligand processes provides a direct tool for exploring important details of DNA structure at the nanoscale. To the best of our knowledge, this is the first report of a comparative AFM and TEM study on the effects of aminoglycoside antibiotics binding on DNA conformation. We have observed some interesting phenomenon of the DNA—drug binding that were not readily noticeable with other standard biochemical techniques. DNA with tobramycin condensed into a variety of complexes such as toroids, rods, and spheres, depending on the ratio of drug-to-base pairs. In addition, many structures were intermediate between the above-mentioned forms. It seems that the condensed conformation of DNA induced by tobramycin results from a specific mode of condensation, rather than by reason of a random assembly of DNA molecules. Moreover, it is known that DNA condensation can be induced under the influence of heavy metal ions (Li et al. 2011). Despite the fact that we used heavy metal stains in TEM study, they have no obvious impact on DNA conformation as compared with the sample without staining (Fig. 6a–c).

Over the last decades, particular interest has been focused on the toroidal structures of condensed DNA (Bloomfield 1997; Dunlap et al. 1997; Golan et al. 1999; Utsuno et al. 2002). For the first time, toroidal condensation of DNA was observed with the use of spermidine as a condensing agent (Gosule and Schellman 1976). During the next years, toroids were examined in detail (Wilson and Bloomfield 1979; Ma and Bloomfield 1994; Bloomfield 1996; Golan et al. 1999). Experimentally, DNA toroids are commonly observed in the presence of multi- and polyvalent cations (Bloomfield 1997). The toroidal condensation of DNA also occurred in the presence of several drugs and dyes. AFM studies of DNA interaction with the minor groove binder netropsin, a natural cationic antibiotic, have indicated that netropsin condenses long λ-DNA molecules into toroids (Adamcik et al. 2008). Another molecule, the minor groove binding dye Hoechst 33258 has been found to induce modifications of DNA structure (Utsuno et al. 2002). It seemed to act as a clamp for DNA molecules, causing condensation of chains and toroid formation. In contrast to groove binders, intercalators induce aggregation of DNA; however, toroidal forms were not observed (Utsuno et al. 2001; Cassina et al. 2011).

It was proposed that DNA condensation is caused by coil-globule transition (Noguchi et al. 1996). This model is based on the assumption that a ring-like form is generated along the chain at first, and afterwards the remaining coiled parts wind around the ring in a gradual way, and a stable toroid is ultimately formed. Moreover, it was suggested that toroids correspond to the minimum energy state and rods are the metastable or kinetically stable state. Another explanation assumes that the toroidal structure may be formed by a self-assembly process in which toroids are created by circumferentially wound DNA primarily or by bending rods (Bloomfield 1991). The first direct observation of toroids allowed to indication that the subunits in toroid are parallel DNA molecules (Dunlap et al. 1997). The structures were formed by folding rather than winding DNA. Two types of toroids with ordered structures caused by spermidine–DNA interaction were observed, such as small toroids with a gap and much larger completely self-connected toroids (Lin et al. 1998).

At least, based on the research works two models for the structure of toroidal condensates were proposed—the spool model and the constant-loop model (Utsuno et al. 2002; Zhou et al. 2013). In both, the axis lies at the center perpendicular to the toroid plane and DNA molecules are wound around it. In the spool model, DNA chains coil parallely without any gap like the threads in a spool and finally end up in a terminal loop. This mechanism is observed primarily in the case of circular plasmid DNA. In the second model, a possible way to arrange DNA molecules includes coiling of chains into equally sized contiguous loops at a vertical section to leave a gap in the center of the toroid. Moreover, many toroids seem to result from the annularization of rods.

Our experimental results suggest the second model and rods opening rather than the first one. It seems that toroids may be an original structure formed directly from DNA, or a derivate product obtained from rods. Similar conclusions were drawn by Utsuno et al. (2002). The high-resolution TEM image (Fig. 5b) shows that the toroid consists of several circumferentially arranged DNA strands, which appear to assemble before forming the toroid. Our work in this area demonstrates that several intermediate forms between toroidal, rod-like, and globular structures can be formed. Figure 4d shows a structure that has the appearance as a complex of toroid with rod. This may suggest that DNA undergoes transformation from one form to another, depending on the tobramycin:DNA ratio. DNA molecules fold several times into rods, which can open up into toroids, and the latter may form spheres with an excess of the drug (Dunlap et al. 1997; Golan et al. 1999).

Previous studies have proposed that free energies such as electrostatic energy, hydration energy, bending energy, and other forms of energy may be the crucial driving forces arranged in DNA condensation (Wilson and Bloomfield 1979; Lin et al. 1998). In our work, DNA packing into condensed structures by tobramycin is probably dominated by electrostatic interactions. Since in solution tobramycin is a multivalent ion with a charge between +3 at pH 8 and +5 at pH 7 in water (Drew et al. 2009), it can result in a large decrease in the repulsion between charged DNA segments. It is known that condensation occurs when ~90 % or more of the charge along the DNA chain is neutralized (Bloomfield 1991; Korolev et al. 2009). Therefore, this may have implications on the fact that the degree of DNA condensation increased progressively (from rods to spheres) as the concentration of tobramycin increased.

Surprisingly, kanamycin and neomycin caused double-strand cleavage and aggregation of some broken chains but did not lead to the condensation of DNA chains. Despite such a high number of studies focusing on aminoglycoside antibiotics, little is known about interactions of kanamycin and neomycin with DNA at the molecular level. To date, only a few studies on the effects of these drugs on DNA have been carried out. Indeed, they showed that kanamycin may affect and cleave DNA chains (Bisset et al. 2004; Kang et al. 2012). Nonetheless, these experiments were performed on living bacteria and the results depended on the presence of specific genes in bacterial genome. On the other hand, neomycin induced thermal stabilization of DNA triple helix, while it had little effect on the stabilization of the B-DNA duplex (Arya and Coffee 2000). Furthermore, double-stranded DNA can be effectively converted from the B-DNA to the A-DNA conformation by this drug (Robinson and Wang 1996). Neomycin was indicated as the most efficient aminoglycoside in cleaving DNA strands at basic sites (Perigolo De Oliveira et al. 2013). As mentioned above, kanamycin also has cleaving activity, however weaker than that of neomycin. Nonetheless, there is an insufficient number of research works on the direct impact of kanamycin and neomycin on native DNA duplex. In our work, we observed that both kanamycin and neomycin have a toxic effect on DNA. The changes in double-stranded DNA structure result from a direct action of aminoglycosides on nucleic acids. However, the above-described interaction did not lead to blocking of DNA molecules, but direct damage of genetic material. Our findings may suggest that this cleavage activity could be related to aminoglycoside toxicity such as a strong ototoxicity or nephrotoxicity, but also to other side effects including the formation of malignant tumors as a result of mutations in the human genome.

This different behavior between tobramycin and kanamycin or neomycin may result from their different chemical structure, which reflects a different binding mechanism with DNA duplex. Tobramycin has one hydroxyl moiety less in the sugar component compared to kanamycin (Fig. 1a, b). On the other hand, neomycin has an additional hexose ring (Fig. 1c). The structural differences cause the different size of hydration spheres of these aminoglycosides. All three aminoglycosides expose their amino groups to the water phase (Bhosale et al. 2006), but only tobramycin may enter the minor groove and cause coiling; kanamycin and neomycin have no apparent effect. The much smaller hydration sphere may thus be responsible for the selective binding of tobramycin in the DNA minor groove. Then, the positive charge of tobramycin neutralizes the negative charge of DNA chains. Furthermore, the deoxyglucose of tobramycin can also directly interact with the deoxyribose walls of the minor groove of DNA. The tobramycin–DNA adduct can then undergo a helix–coil transition, which takes care of the increased overall volume of the complexed DNA strand (Imai et al. 2001). The model is in perfect agreement with models of tobramycin–DNA complexes within hydrophobic environments: the diamines and the deoxy site of the first unit steer the tobramycin onto two phosphate units (Drew et al. 2009); the axial hydrogens of the remaining two sugars provide the hydrophobic site comparable to immobilized d-glucose units in hydrophobic yoctowells (Bhosale et al. 2006).

## Conclusions

The interactions of aminoglycoside antibiotics tobramycin, kanamycin, and neomycin with calf thymus DNA were examined at the monomolecular level using three different experimental techniques, supported by molecular simulations. We observed significant differences between their mechanisms of interaction with the DNA duplex. Tobramycin was found to induce the condensation of nucleic acids into complex structures, which subsequently results in blocking of biological activity of DNA. In contrast, kanamycin and neomycin gave rise to direct damage of genetic material. The different behavior of tobramycin with respect to other aminoglycosides is likely due to different hydration properties and interaction volumes of carbohydrates and deoxy derivatives presented in the structure of aminoglycoside antibiotics. The hydration sphere of tobramycin is smaller, therefore the drug may interact with the minor groove of the DNA duplex. Studies on the mechanisms by which small molecules interact with DNA are important in understanding their functioning in cells, designing of new and efficient drugs, or in minimizing their adverse side effects. Our results have demonstrated the condensation effects of aminoglycosides on genetic material. This phenomenon may lighten the ototoxicity or nephrotoxicity issues, but also other adverse reactions of aminoglycoside antibiotics in human body.

## Electronic supplementary material

Below is the link to the electronic supplementary material.

Supplementary material (MPG 14,590 kb)
